# Phase-pure ferroelectric quantum wells with tunable photoluminescence for multi-state optoelectronic applications

**DOI:** 10.1038/s41377-025-01874-2

**Published:** 2025-06-30

**Authors:** Rui Sun, Yuping Jia, Bo Lai, Zhiming Shi, Mingrui Liu, Weili Yu, Ke Jiang, Shanli Zhang, Shunpeng Lv, Yang Chen, Xiaojuan Sun, Dabing Li

**Affiliations:** 1 Key Laboratory of Luminescence Science and Technology, Chinese Academy of Sciences & State Key Laboratory of Luminescence and Applications, Changchun Institute of Optics, Fine Mechanics and Physics, Chinese Academy of Sciences, Changchun, China; 2 University of Chinese Academy of Sciences, Beijing, China

**Keywords:** Optical materials and structures, Lasers, LEDs and light sources

## Abstract

Quasi-two-dimensional (quasi-2D) metal halide perovskite (MHP) ferroelectrics, characterized by spontaneous polarization and semiconducting properties, hold promise for functional photoferroelectrics in applications such as optical storage and in-memory computing. However, typical quasi-2D perovskite films contain multiple quantum wells with random width distribution, which degrade optoelectronic properties and spontaneous polarization. Here, we introduce phase-pure quantum wells with uniform well width by incorporating the inorganic salt MnBr_2_, which effectively controls crystallization kinetics and restricts the nucleation of high n-phases, producing high-quality films. The resulting (BA)_2_CsPb_2_Br_7_ (BA = C_4_H_9_NH_3_) film demonstrates ferroelectric hysteresis behavior, clear in-plane ferroelectric domain switching, and a high photoluminescence quantum efficiency (PLQE) of 88.7%. Significantly, we observed a nonvolatile, reversible in situ photoluminescence (PL) modulation of Mn^2+^ in this ferroelectric MHP film under an applied electric field, attributed to lattice distortion from ferroelectric polarization orientation. These findings enabled the development of a simple system comprising gallium nitride (GaN) light emitting diodes (LEDs) and ferroelectric films to implement multi-state signal encoding and a logic AND gate. This work advances the fabrication of efficient ferroelectric MHP films and highlights their potential for advanced optoelectronic applications.

## Abstract (web summary)

Incorporating MnBr_2_ into (BA)_2_CsPb_2_Br_7_ films enhanced ferroelectric properties, alongside a high PLQE of 88.7%, achieving a nonvolatile, reversible in situ photoluminescence modulation of Mn^2+^.

## Graphical abstract

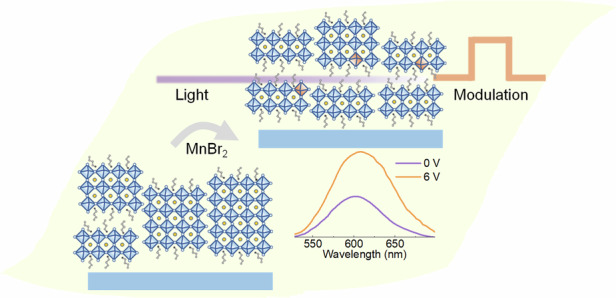

## Introduction

Ferroelectrics exhibit the characteristic of switchable spontaneous electrical polarization and have long been of great interest in nanoelectronics and nonvolatile device applications^1–4^. Coupling ferroelectricity with optical properties in semiconductors holds great promise for enhancing the material functionality and giving rise to new physical phenomena, enabling the development of optical switches, photoelectric modulators, in-memory computing and optical storage devices^5–9^. These devices necessitate materials that possess both ferroelectric properties and high luminescence efficiency. Conventional oxide perovskite ferroelectrics show the excellent ferroelectric properties^10,11^. However, the application scope of oxide perovskite is constrained by their insulative characteristics, coupled with low charge carrier mobility, substantial bandgap (3–5 eV), and absence of luminescent properties.

Metal halide perovskites (MHPs) are structurally analogous to oxide perovskites, but represent a new class of semiconductors, distinct from insulating oxide counterparts. Due to superior optoelectronic features, such as high mobility, large absorption cross section, and adjustable bandgap, MHPs have attracted a lot of attention^12–14^. In particular, the integration of perovskite with gallium nitride (GaN) light-emitting diodes (LEDs), combined with the stability and high output power of GaN LEDs^15–17^, has the advantages of high efficiency, high color purity and low cost, shows broad application prospects in the field of optical communications^18,19^. Among the broad types of MHPs, quasi-two-dimensional (quasi-2D) MHPs, exhibit spontaneous polarization due to the synergistic effect of the ordered arrangement of organic components and the relative displacement of the octahedra, which are attractive candidates for novel semiconductor ferroelectrics^20–22^. Currently, most reported quasi-2D ferroelectrics are bulk crystals, whose growth process is time-consuming, uncontrollable, and small in scale^23–25^. On the other hand, the bulk ferroelectric crystals are unable to meet the increasing demand for high-performance and multifunctional devices. Therefore, the growth of quasi-2D MHP ferroelectric films has become a key research focus. As far as we known, the random well-width distribution during the crystallization of quasi-2D MHP films will significantly reduce the photoelectric performance of the devices, as well as the spontaneous polarization. Certain methodologies aimed at augmenting the photoelectric performance have centered on the elimination of low n-phases to achieve a more uniform distribution of high n-phases, a strategy that impedes the realization of ferroelectricity ^26–29^.

Homogeneous crystallization is the prerequisite to guarantee high-quality quasi-2D film formation. Herein, we introduced manganese bromide (MnBr_2_) to regulate the growth dynamics of perovskite by changing the pH value of solvent. The resulting film (BA)_2_CsPb_2_Br_7_ with MnBr_2_ (BA = C_4_H_9_NH_3_) added demonstrates a low n-phase pure distribution and the photoluminescence quantum efficiency (PLQE) increased from 11% to 88.7%. Macroscopic polarization hysteresis loop and piezoelectric force microscopy measurements confirmed the existence of ferroelectricity with in-plane ferroelectric domains. Furthermore, we demonstrate nonvolatile and reversible electrical modulation of PL from Mn^2+^ under an electric field. The modulation is the result of lattice distortion caused by the orientation of ferroelectric polarization along the electric field. These findings empower the implementation of multi-state signal encoding and logic AND gate operations within a straightforward system comprising GaN LEDs and ferroelectric films. These results enable us encode the digital signal into PL intensity of Mn^2+^, demonstrating multi-state signal encoding and logic AND gate functions in proof-of-concept devices. We anticipate that this study will open a pathway for further exploration into this class of semiconducting ferroelectric materials and trigger new possibilities in developing newly multi-functional materials.

## Results

**Fig. 1:**
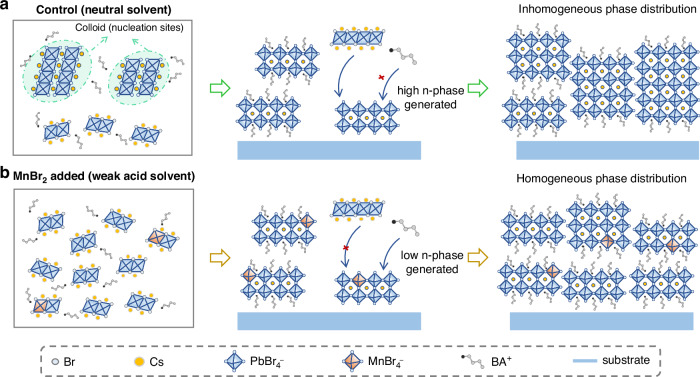
**Schematic diagram for nucleation and growth processes.** **a**, **b** The nucleation and growth processes of (BA)_2_CsPb_2_Br_7_ MHP films without and with MnBr_2_ added. **a** The cyan dotted circle represents colloidal precursor sol-gel Cs^+^/PbBr_x_^−^ complexes, which formed spontaneously in the precursor solution. The colloids provided nucleation sites for the subsequent crystallization. **b** The incorporation of MnBr_2_ changed the solvent acidic, dissolving the colloids in the perovskite precursor solution. The cations are evenly distributed in the precursor solutions

The perovskite precursor solutions comprised of CsBr, PbBr_2_, BABr, and a varying ratio of MnBr_2_ in DMSO (see Methods for details). The ratio of added MnBr_2_ is defined as the molar ratio of MnBr_2_ to PbBr_2_ (hereinafter referred to as “x% MnBr_2_”). Corresponding quasi-2D films were fabricated by one-step spin-coating process in a nitrogen-enriched environment. Regulating the growth kinetics is a key to achieving a pure low n-phase distribution in quasi-2D perovskite films. Typically, perovskite precursor solutions spontaneously form colloids that serve as nucleation sites, thereby accelerating the local crystallization rates. As shown in Fig. 1a, lower n-phases show higher preferred crystallinity because they have a lower crystallization energy barrier compared to higher n-phases^30^. As a result, the significant consumption of organic cations (BA^+^) has led to a shortage of local organic cations. Therefore, higher n-phases precipitate sequentially, resulting in a broader phase distribution. According to previous research, the colloid concentration can be manipulated by adjusting the pH value of the solution^31^. With this in mind, we introduced manganese bromide into the precursor solvent to regulate the pH (Table S1), where MnBr_2_ results in an increase in acidity (or decrease in basicity) to suppress the colloids’ formation (Fig. 1b), leading to homogeneous and narrow lower n-phase distributed quasi-2D films.

**Fig. 2:**
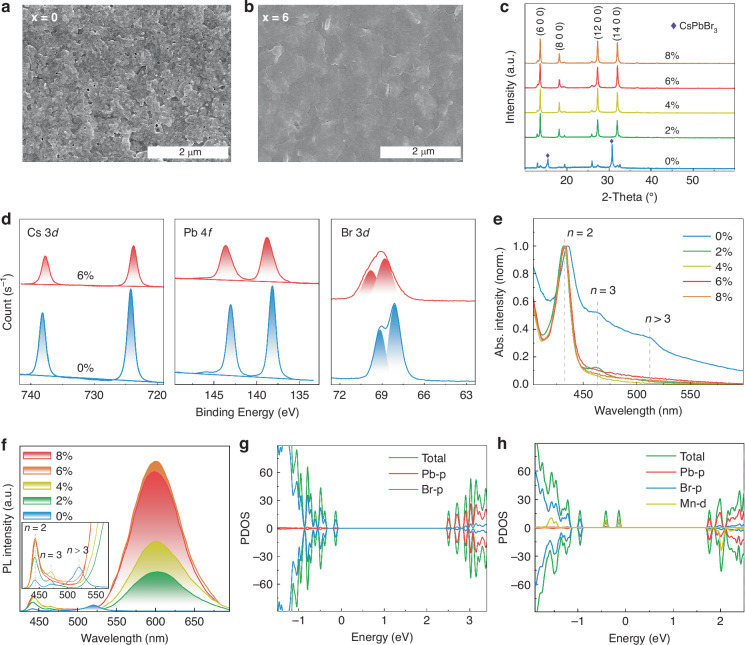
**Structural and optical characterizations.** **a**, **b** SEM images of pristine film and 6% MnBr_2_ added film, respectively. **c** XRD patterns of the 0–8% MnBr_2_ added films. **d** XPS spectra of pristine and 6% MnBr_2_ added films for the binding energy regions corresponding to the Cs 3d, Pb 4f, Br 3d. **e**, **f** UV-vis absorption spectra, PL spectra of the 0–8% MnBr_2_ added films (inset is the local magnification of the PL spectra). **g**, **h** PDOS of pristine film and 6% MnBr_2_ added film, respectively

Scanning electron microscopy (SEM) measurement was used to investigate the film morphology, as shown in Fig. 2a, b and Fig. S1. All films exhibit complete substrate coverage, with those containing 6% and 8% MnBr_**2**_ demonstrating minimal pinholes and the most densely packed grain structures. To access the influence of MnBr_2_ on film crystallinity and phase composition, X-ray diffraction (XRD) measurement was conducted (Fig. 2c). The pristine film exhibits diffraction peaks at 15.4° and 30.7°, which can be assigned to 3D CsPbBr_3_ phase. And the other peaks match well with 2D MHP phase^32^. Upon introducing MnBr_2_, the 3D diffraction peaks disappeared. More importantly, all the main diffraction peaks in the MnBr_2_ added films correspond to (h 0 0) planes, indicating that the films have a highly-oriented arrangement perpendicular to the crystallographic a-axis direction. To further investigate the distribution and crystal structure of films, the transmission electron microscopy (TEM) measurement was performed. Obviously, the incorporation of MnBr_2_ change the basic morphology of MHP films (Fig. S2a). It was magnified in Fig. S2b, (6 0 0) and (12 0 0) planes of quasi-2D orthorhombic (BA)_2_CsPb_2_Br_7_, and (1 0 0) plane of 3D cubic CsPbBr_3_ can be observed in pristine film. After incorporating MnBr_2_, only (6 0 0) and (12 0 0) planes of quasi-2D perovskite remained, further proves that MnBr_2_ promoted the growth of 2D phase and inhibited the formation of 3D phase. The X-ray photoelectron spectroscopy (XPS) test on pristine and 6% MnBr_2_ added films was used to further confirm the chemical states. The binding energy (E_b_) of Pb-4f and Br-3d shift to larger E_b_, and that of Cs 3d shift toward smaller E_b_ (Fig. 2d). Such slight shift toward higher E_b_ probably originates from the decreased Mn-Br distance, which results in the distortion of the [Pb(Mn)Br_6_]^4−^ octahedra^33^. Additionally, the Mn 2p signals at 653.1 eV and 641.3 eV are observed in 6% MnBr_2_ added (BA)_2_CsPb_2_Br_7_ film (Fig. S3), confirming the presence of Mn^2+^. Fig. S4 provides the atomic force microscopy (AFM) images of the pristine and 6% MnBr_2_ added films. The root-mean-square (RMS) of the films across a scanned area of 1 × 1 μm^2^ were determined to be 12.2 and 9.9 nm, respectively, suggesting the superior film quality of 6% MnBr_2_ added film.

We further conducted comparative studies on the optical properties of pristine and MnBr_2_ added films. UV-vis absorption spectra show the variation of n-phase distribution in the as-prepared films (Fig. 2e). The pristine film displays one dominant absorption peak at 435 nm and two obvious absorption signals at 465 and 512 nm, belonging to n = 2, 3, and >3 phases, respectively. In contrast, the 2% MnBr_2_ added film shows absorption signals concentrated on the n = 2 and 3 phases, and the x > 2-samples exhibits only one absorption peak at 435 nm (n = 2). These results can be concluded that the introduction of MnBr_2_ promotes the formation of the lower n-phase and improves crystallinity. As is known to all, variations in n-phase distribution will lead to different emission peaks^34^. The PL measurement was performed to further confirm the effect on n-phase distribution (Fig. 2f). The pristine film exhibits three emission peaks at 443 nm, 470 nm, and 520 nm, corresponding to n = 2, 3, and >3 phases, respectively. The MnBr_2_ added films show only two exciton emission peaks located at n = 2, 3, and a broad orange emission peak centered at 600 nm comes from the ^4^T_1_ to ^6^A_1_ transition of Mn^2+^. As x increases, the emission intensity of Mn^2+^ obviously increases. However, for the samples with 8% MnBr_2_ added, the emission of Mn^2+^ decreases sharply, which may be caused by concentration quenching.

We further performed first-principles density functional theory (DFT) calculations to understand the band structure. The partial density of states (PDOS) of both pristine and 6% MnBr_2_-added films are shown in Fig. 2g, h. These DFT calculations were conducted using the Vienna Ab initio Simulation Package, which is based on the pseudopotential plane-wave method. The introduction of MnBr_2_ increases the optical bandgap from 2.405 eV to 2.474 eV, which is consistent with the absorption data. As shown in Fig. 2g, in the pristine film, the valence band (VB) is primarily contributed by Br p orbitals, while the conduction band (CB) is mainly contributed by Pb p and Br p orbitals. In contrast, in the PDOS of the MnBr_2_ added film (Fig. 2h), Mn has minimal influence on the edge states. Additionally, two spin-up mid-gap bands are observed in the bandgap, and one spin-down mid-gap band is found just below the conduction band minimum. However, these mid-gap states exert little influence on the radiative recombination of excitons.

**Fig. 3:**
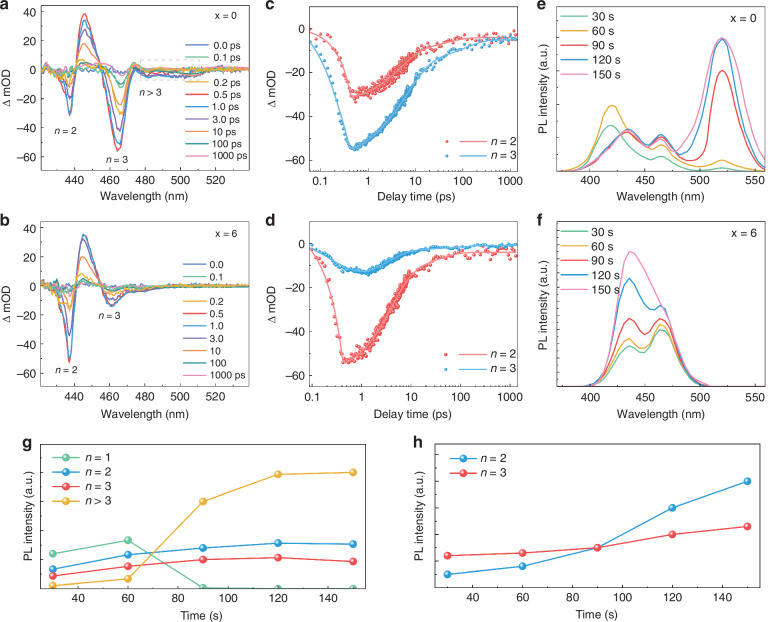
The phase distribution regulated by MnBr_2_. **a**, **b** TAS under different delay times for pristine and 6% MnBr_2_ added films, respectively. **c**, **d** TA dynamic curves and fitting results of *n* = 2 and *n* = 3 phases in pristine and 6% MnBr_2_ added films, respectively. **e**, **f** Time varying in situ PL spectra of pristine and 6% MnBr_2_ added films. **g**, **h** The variation of PL intensity of different n-phase over time, which are the integral results of (**e**, **f**)

In addition, femtosecond transient absorption spectroscopy (TAS) measurements were conducted to investigate the phase distribution (Fig. 3a, b). The pristine film initially exhibits two distinctive bleaching peaks at 437 nm and 463 nm, corresponding to n = 2 and 3 phase, respectively (Fig. 3a). The two bleaching peaks show a slight redshift (around 1 nm), commensurate with the appearance of an n > 3 bleaching peak at a longer wavelength (from 474 nm to 512 nm) after 0.5 ps. In contrast, TAS for 6% MnBr_2_ -containing film shows bleach peaks at 437 nm and 463 nm, corresponding to n = 2, 3 phase, whose characteristics are stable over the time range of 100 ps (Fig. 3b). The TAS of 6% MnBr_2_ added film shows a reduction in the number of bleaching peaks, which demonstrates that 6% MnBr_2_ regulate the phase distribution of quasi-2D MHP films. The enhancement of bleaching peak intensity of n = 2 phase at 1 ps in 6% MnBr_2_ added film implies an increase in the amount of the n = 2 phase, which is consistent with the absorption spectra and XRD results. Then we plotted the absorption curves when the delay time between the pump and probe light is 1 ps, during which the intensity of photobleaching (PB) peaks represents the populations of corresponding phases. The relative content of different phases is further calculated by integrating these PB peaks and the results are shown in the histogram (Fig. S5). In the pristine film, severe segregation in the n-phase distribution is observed, where n = 2, 3 and n > 3 phases account for the major part. As MnBr_2_ incorporated, the amount of n ≥ 3 phase sharply decreased, proving that MnBr_2_ plays a role in regulating the phase distribution of MHP films. In addition, the pristine film exhibits the ultrafast decay component of τ_1_ = 0.48 and 0.42 ps (n = 2 and 3), respectively, whereas 6% MnBr_2_ added film shows a relatively slower decay time of τ_1_ = 0.95 ps for n = 2 phase and a relatively faster decay time of τ_1_ = 0.23 ps for n = 3 phase, respectively (Fig. 3c, d and Table S2). These results demonstrate convincingly that the incorporation of MnBr_2_ promotes the generation of n = 2 phase and inhibits the n ≥ 3 phases of the films.

Apparently, incorporating MnBr_2_ could regulate the phase distribution of films. In order to study the action mechanism of MnBr_2_, we employed in situ PL measurement to analyze the growth kinetics of (BA)_2_CsPb_2_Br_7_ MHP films. The precursor solution was spin-coated onto the substrate and the PL spectra were collected during the evaporation of solvent. The PL spectra with DMSO solvent evaporation for pristine and MnBr_2_ added films were shown in Fig. 3e, f, respectively. The pristine film showed more complex phase composition than MnBr_2_ added film. To better demonstrate the variation of phase distribution with solvent evaporation time, we integrated the emission peaks in PL spectra and calculate the proportion of each n phase to the total emission area, and obtained the curve as shown in Fig. 3g, h. We clearly observed that n = 1, n = 2 and n = 3 components in the initial pristine film exhibits stronger PL intensity. After 90 s, the n = 1 component began to decrease as n = ∞ component increasing. The color of the film gradually changes from blue to green (Fig. S6a). This indicates that the 2D perovskite was first formed during crystal growth, and the n = ∞ composition, which is quite close to 3D perovskite, generated gradually as extra cations were inserted into the 2D framework. In contrast, only n = 2 and n = 3 components exist, and the PL intensity of n = 2 phase gradually dominates. The color of the film remains blue (Fig. S6b). The results indicate that MnBr_2_ successfully alters the growth kinetics of the film and avoids the broad phase distribution, which is consistent with our original hypothesis.

Then, we analyzed the PL uniformity of the films using a confocal fluorescence microscopy imaging system (Fig. S7). The PL of 6% MnBr_2_ added film is significantly denser and stronger than that of pristine film. The corresponding PLQE increased from 11% for the pristine film to 88.7% when 6% MnBr_2_ was added. It was also found that too much MnBr_2_ added resulted in a decrease in PLQE (Figs. S8, 9). To ensure the reliability of these results, we also tested samples from different batches (Fig. S10), and the results consistently showed that the addition of MnBr_2_ significantly enhanced PLQE. Then, the trap densities of the MHP films were measured via a space charge limited current (SCLC) methods^35,36^ by fabricating both hole-only devices and electron-only devices (Fig. S11), which were calculated according to the following equation: *N*_*t*_ = (2εε_0_V_TFL_)/(eL^2^) where ɛ_0_ is the vacuum permittivity, ɛ is the relative dielectric constant, V_TFL_ is the trap-filled limit voltage, e is the electron charge, and L is thickness of the film. The trap densities in the 6% MnBr_2_ added films are *N*_*t*_(e) = 1.3 × 10^15^ cm^−3^ and *N*_*t*_(h) = 1.5 × 10^15^ cm^−3^ (where the subscripts “e” and “h” represent electrons and holes, respectively), which are approximately half those of the pristine film (Table S3). The fall-off in trap state densities indicates the better quality of film, agreeing with the results of XRD measurement. The long-term measurements of the environmental and thermal stability of the films were then carried out (Fig. S12). When the films stored in atmospheric conditions, the PL of pristine film decayed to 60% of original value within 30 days. While the 6% MnBr_2_ added film retained more than 60% of initial PL intensity for 80 days. Similarly, the PL of pristine film decayed by more than 40% of initial value when heated at 80 °C for 30 days, whereas 6% MnBr_2_ added film shows less than 20% decay. We believe that the better stability of 6% MnBr_2_ added film is due to its high crystallinity and uniform phase distribution^37,38^. Time-resolved photoluminescence (TRPL) decay processes of Mn^2+^ are shown in Fig. S13. The fitted decay constant varies from 708.9 μs to 899.1 μs as the MnBr_2_ content changes from 2% to 8%, which again suggest the emission originated from Mn^2+^ ions.

**Fig. 4:**
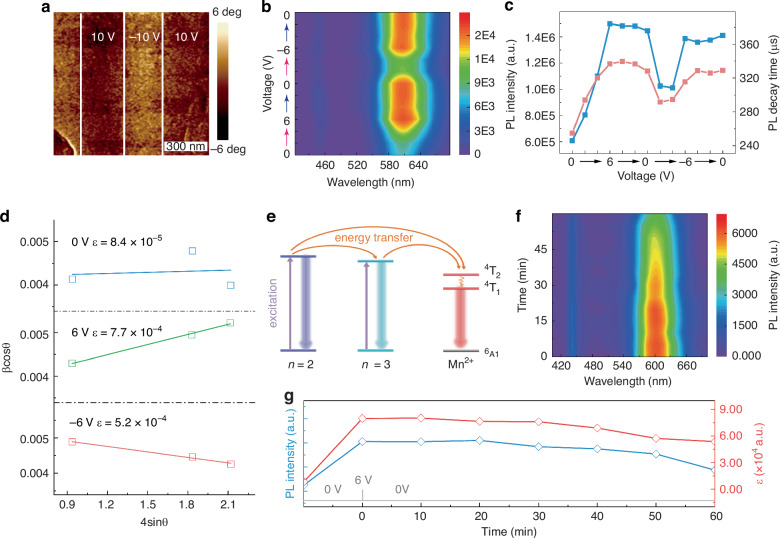
**Ferroelectricity regulates the luminescence.** **a** Lateral PFM phase image of the after applying a tip voltage *V* = ± 10 V to the 6% MnBr_2_ added film. **b** Contour plot of voltage-dependent PL spectra. **c** The PL intensity and PL decay time of Mn^2+^ as a function of applied voltage, respectively. **d** W-H plots of 6% MnBr_2_ added film under 0, ±6 V voltage. **e** Schematic illustration of the energy transfer mechanism. **f** Time-dependent PL intensity after the applied pulse voltage was withdrawn. **g** PL intensity and lattice distortion (*ɛ*) as a function of applied voltage, respectively

The phase purity of the quasi-2D MHP films ensures their ferroelectric properties^39^. To confirm the ferroelectricity of 6% MnBr_2_ added film, a polarization-voltage (P-V) hysteresis loop was carried out (Fig. S14). The curve shows typical ferroelectric behavior with both residual polarization and a strong coercive voltage. The P-V hysteresis loop determines the macroscopic manifestations of the ferroelectric properties. Piezoelectric force microscopy (PFM) technology has opened up avenues for exploring microscopic ferroelectricity by imaging and manipulating polarization vectors or ferroelectric domain at nanoscale. Fig. S15 present the PFM phase images of the film, exhibiting uniform contrast throughout. By applying a constant voltage V = ± 10 V to the tip and scanning the film surface, we poled different regions of film (Figs. 4a and S16). The vertical phase image shows that the out-of-plane domain switching is absent before and after the application of the electric field (Fig. S16). The lateral phase contrast depicted in Fig. 4a distinctly delineates two regions with opposing polarizations, confirming that the polarization is entirely confined within the plane of the film.

Due to the excellent optical and ferroelectric properties, the (BA)_2_CsPb_2_Br_7_ film with 6% MnBr_2_ added was incorporated into the light modulation device with the structure of ITO/MHP film/Ag, and we designed a system to detect the voltage-dependent PL spectra/ PL intensity (Fig. S17). The voltage-dependent PL measurement is shown in Fig. S18. For a more intuitive presentation, Fig. 4b presents a contour plot of the PL spectra under varying voltages, highlighting the substantial variation in the orange emission resulting from the ^4^T_1_ to ^6^A_1_ transition. Obviously, the PL can be strongly enhanced when increasing the voltage, with the enhancement factor reaching up to 2.47 (Fig. S19). Notably, the applied voltage adheres to a hysteresis loop pattern with increments of 2 V. The blue curve in Fig. 4c summarizes the PL intensity versus the applied voltage. As the voltage increases from 0 V to 6 V, the PL intensity also increases. During the voltage decrease from 6 V back to 0 V, the PL intensity remains relatively stable. When the voltage shifts from 0 V to −6 V and back to 0 V, the rotation of PL intensity is similar to that observed for positive voltage. The corresponding variation of PL decay dynamics with voltage is consistent with PL intensity (Fig. S20, pink curve in Fig. 4c), indicating alterations in the local crystal field environment of Mn^2+^ ions. The in situ PL enhancement is believed to stem from the electric field-induced increment in lattice distortion, which is confirmed by the variation in lattice distortion under different applied voltages. Williamson-Hall (W-H) analysis was introduced to analyze the variation of lattice distortion for the sample as a function of bias voltage^40,41^. The slope (*ɛ*) in W-H plot qualitatively represents the degree of lattice distortion in the film. As shown in Fig. S21, Fig. 4d, and Table S4, the *ɛ* value of the film with applied voltage is significantly higher than that of the film without applied voltage, meaning that the electric field leads to an increase in the lattice distortion of the film.

Figure 4e illustrates the energy levels and possible carrier relaxation pathways in the MnBr_2_ added (BA)_2_CsPb_2_Br_7_ films. First, the excitons of n = 2, 3 phase hosts can be photogenerated upon UV excitation. The excitons of n = 2 phase can transfer energy to n = 3 phases and the excited state of Mn^2+^ dopants. Then the excitons from n = 3 phases can also transfer energy to Mn^2+^ ions. Under the electric field, the lattice deformation leads to enhanced exciton-phonon coupling, making it easier for Mn^2+^ ions to relax from the excited state to the ground state, resulting in enhanced luminescence of Mn^2+^^42^. Moreover, we observed a PL keeping phenomenon in the film under applied voltage. The nonvolatile PL state was confirmed by imposing a voltage pulse (Fig. 4f). Before the PL measurement, a voltage of 6 V was applied, the time-dependent PL intensity of Mn^2+^ was summarized in Fig. 4g. After turning off the bias voltage, the PL intensity remained almost unchanged within 30 min and then decreased with increasing time. The corresponding lattice distortion degree shows the same change trend.

**Fig. 5:**
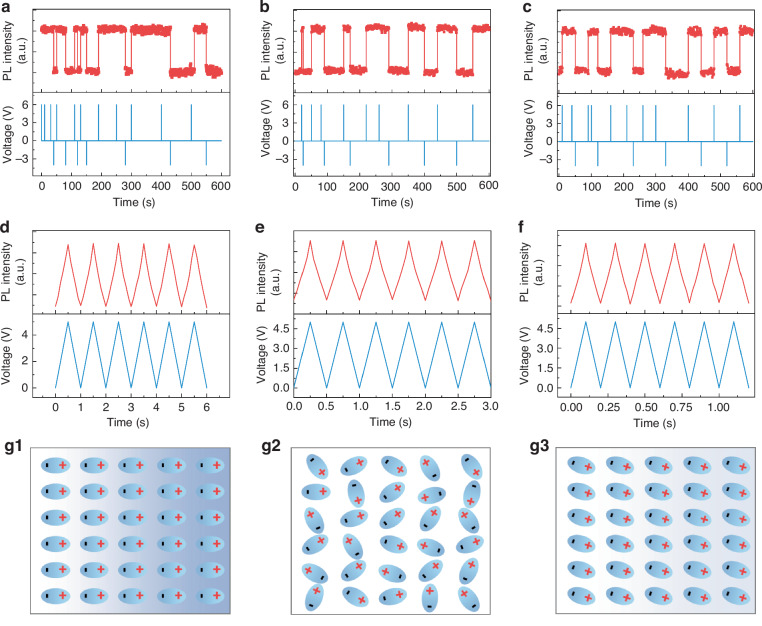
**Voltage-dependent PL intensity.** **a**–**f** PL intensity modulation of the 6% MnBr_2_ added film by imposing pulse voltage with different pulse widths. The lower curve represents the applied pulse voltage, while the upper curve shows the variation of PL intensity with the applied voltage. **g** Diagram of electric field modulation of PL intensity, where fig. g2 is the initial ferroelectric polarization state, fig. g1 and fig. g3 show the ferroelectric polarization states with completely and incompletely switched, respectively

Based on the electrically adjustable PL intensity of the film, we conducted PL measurement by applying voltage pulses of varying pulse widths on the film. As illustrated in Fig. 5a–c, a voltage with pulse widths of 0.25–1 s, the amplitude of 6 V for the “ON” state and −4 V for the “OFF” state was employed to induce reversible and nonvolatile PL intensity states. When the pulse width was reduced below 0.1 s (Fig. 5d–f), the PL intensity was observed to be modulated synchronously with the applied voltage. To elucidate the mechanism, Fig. 5g illustrates the possible variation of ferroelectric polarization under the applied electric field. The initial ferroelectric polarization is out of order in the absence of external electric field (Fig. 5g2). When voltage with 0.25–1 s pulse width was applied, the ferroelectric polarization states were completely switched to the orientation of along the direction of the electric field by imposing positive voltage, and this polarization state can be remained after removing the voltage (Fig. 5g1). And a small negative voltage can erase this polarization state. In contrast, the voltage with shorter pulse widths (0.02–0.1 s) causes the incomplete polarization switching, the PL intensity cannot be remembered (Fig. 5g3).

**Fig. 6:**
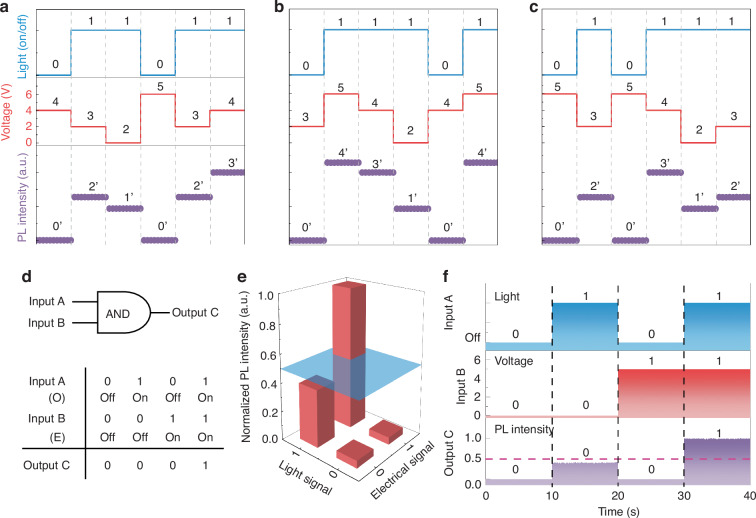
**Multi-state signal encoding and logic AND gate functions in proof-of-concept devices.** **a**–**c** Optoelectronic communication demonstration of PL intensity modulation devices. From top to bottom, the switch of the GaN LED and the applied voltage are used as input signals, and the monitored PL intensity is used as the output signal. **d**–**f** Working principle and demonstrations of logic AND gate. Schematic and operating principle of logic AND gate (**d**). 3D bar plot of the normalized PL intensity of 6% MnBr_2_ added film (**e**). The normalized PL intensity above and below 0.5 threshold is considered as output codes “1” and “0”, respectively. The dynamic demonstration of logic AND gate (**f**)

Five distinct levels of PL intensity were obtained via optical/electrical dual modulation, as shown in Fig. S22. All intensity states can be easily distinguished. The impressive nonvolatile modulating and multi-state memory characteristics empower the perovskite films for multi-state signal coding (Fig. 6a–c). During operation, the on/off of the excitation light source GaN LED is used as input signals “0” and “1”. And the applied voltages of 0, 2, 4 and 6 V corresponding to the “2”, “3”, “4”, “5” input signals, respectively. By combining the input optical and electric signals, five PL intensity states can be generated, labeled as “0′”, “1′”, “2′”, “3′” and “4′”, respectively. Ultimately, 5^5^ total information states can be achieved within the simple structure. Besides visualizing signal coding as mentioned above, we also constructed an opto-electronic logic gate device. In the proposed logic device, a pair of optical and electric digital serve as two independent inputs (O_in_ and E_in_) to control the output PL signals (P_out_). Figure 6d shows the diagram of the logic AND gate and their truth table. In this process, the optical signal was generated by a GaN LED chip as the input signals of “0” and “1”. And the applied voltage of 0 V and 5 V is considered as the input electrical signal of “0” and “1”, respectively. The schematic illustration of the AND gate operating principle was described in Fig. 6e, where four separate intensities are generated based on four combinations of input signals. All intensities are normalized to the PL intensity under the condition of optical signal on and a 5 V voltage. And the logic outputs TRUE (1) when the normalized value is higher than 0.5, and FALSE (0) when the normalized value is lower than 0.5. Finally, we perform the dynamic demonstration of the logic AND gate (Fig. 6f), where the expected logic outputs are extracted synchronously.

## Discussion

In summary, we have elucidated the critical role of homogeneous phase distribution in the fabrication of quasi-2D MHP ferroelectric films, and developed a novel strategy to regulate crystallization kinetics to realize low-n pure phase distribution by incorporating MnBr_2_. The approach effectively addresses the challenge of broader phase distribution, rendering the production of high-quality, high photoluminescence quantum efficiency quasi-2D ferroelectric films feasible. Both P-V loop and PFM measurements confirmed the ferroelectric feature in the film. Consequently, the nonvolatile in situ PL modulation of Mn^2+^ was demonstrated through local application of electric field. The PL modulation can be attributed to the fact that the orientation of ferroelectric polarization along the electric field direction facilitates the lattice distortion. Based on controllable and nonvolatile PL modulation, multistate memory and logic functions were realized through encoding the excitation light signal with the electric signal through a sample system. Our results offer an effective approach for preparing high-performance quasi-2D MHP ferroelectric films and provide strategies for further exploration of new ferroelectrics for diverse optoelectronic applications.

## Materials and methods

### Materials

Lead bromide (PbBr_2_), Cesium bromide (CsBr), butylammonium bromide (BABr), Manganese bromide (MnBr_2_), dimethyl sulfoxide (DMSO) and chlorobenzene (CB) were purchased from Alfa Aesar. All the chemicals are used directly without further purification.

### Perovskite film and device fabrication

The precursor solution is prepared with equation of (BA)_2_CsPb_2_Br_7_ with x% MnBr_2_ added. The ITO-coated glass substrates were cleaned by sonication sequentially in detergent, distilled water, acetone, and isopropanol. Then, the substrates were treated in ultraviolet ozone for 30 min. Afterward, the substrates were transferred into the nitrogen-filled glove box and the perovskite precursor was on the top of substrate at 4000 rpm for 40 s. 100 µL CB was poured onto film at 25 s after the spin-coating started. The perovskite films were annealed at 60 °C for 10 min, and then 130 °C for 20 min. Cool naturally to room temperature. Silver was then deposited by thermal evaporation in a vacuum deposition chamber (1 × 10^−7^ Torr).

### Characterizations

The pH value was tested through PHSJ-6L. SEM images were collected in secondary electron mode by a Hitachi S-4800. Powder X-ray diffraction was measured on a Bruker D8-advance X-ray diffractometer with Cu Kα radiation (λ = 1.54178 Å). The TEM images of the MHP films were made on a FEI Tecnai G2 F30 at 300 KeV. AFM images and RMS were collected by Bruker Multimode 8. X-ray photoelectron spectroscopy (XPS) was carried out in a Kratos Axis Ultra DLD spectrometer equipped with a monochromatic Al K_α_ X-ray source (hν = 1486.6 eV) operated at 150 W with a multichannel plate, and a delay line detector under 1.0 × 10^−9 ^Torr vacuum. UV-vis absorption spectra were obtained by using an Ocean Optics and the Maya-2000 fluorescent fiber optic spectrometer. Photoluminescence spectra were measured by Edinburgh-FLS 980 spectrophotometer. Photoluminescence quantum efficiency was then calculated by using the Edinburgh-FLS 980 software package. The confocal fluorescence microscopy images were measured by OLYMPUS FV-1000. Femtosecond pump-probe transient absorption (TA) measurements were performed on a Helios (Ultrafast systems) spectrometers using a regeneratively amplified femtosecond CaF_2_ crystal laser system (Spitfire Pro-F1KXP, Spectra-Physics; frequency, 1 kHz; max pulse energy, ~4 mJ; pulse width, 120 fs). The luminescent dynamics were measured by the Hamamatsu R9110 PMT single-photon counting system. The in situ PL measurement was carried out by Photo Research Spectra Scan spectrometer PR650. PL The P-V hysteresis loop was measured with a ferroelectric measurement system at 1 Hz. The PFM images were measured by a Bruker Multimode 8 equipment, in which the PFM tip applied ±10 V voltages to scan bar region, causing different regions to undergo different polarization inversion process. All the variable voltage experiments use Keithley 2400 source meter to provide voltage.

### DFT calculations

DFT calculations were carried out with the Vienna ab initio simulation package (VASP). The Kohn−Sham equations were solved using a plane wave basis set with an energy cutoff of 500 eV, and the projector augmented-wave (PAW) potential was applied. The crystal structure was optimized using the Perdew−Burke−Ernzerhof exchange-correlation functional with spin polarization. The van der Waals interaction is also included in the calculations. A 2 × 2 × 1 supercell for the 2-layer tetragonal-phase 2D perovskite was adapted in the simulations. The vacuum slabs thicker than 15 Å to avoid artificial interaction of periodic images. We employ a Monkhorst–Pack k-grids with the density of 0.04 2π/Å in the Brillouin zone. In addition, the force and total energy convergence thresholds are set to 0.02 eV Å^−1^ and 10^−5^ eV, respectively.

## Supplementary information

supplementary information

## Data Availability

All data generated or analyzed during this study are included in the published article and its Supplementary Information. Additional data are available from the corresponding author upon request.

## References

[1] Bian, R. J. et al. Developing fatigue-resistant ferroelectrics using interlayer sliding switching. *Science* **385**, 57–62 (2024). 38843352 10.1126/science.ado1744

[2] Wang, N. et al. Molecular engineering regulation achieving out-of-plane polarization in rare-earth hybrid double perovskites for ferroelectrics and circularly polarized luminescence. *Angew. Chem. Int. Ed.* **63**, e202409796 (2024). 10.1002/anie.202409796 38958031

[3] Xie, Z. W. et al. Nonvolatile and reconfigurable two-terminal electro-optic duplex memristor based on III-nitride semiconductors. *Light Sci. Appl.* **13**, 78 (2024). 38553460 10.1038/s41377-024-01422-4 PMC10980680

[4] Liu, M. R. et al. Effect and regulation mechanism of post-deposition annealing on the ferroelectric properties of AlScN thin films. *ACS Appl. Mater. Interfaces* **16**, 16427–16435 (2024). 38523333 10.1021/acsami.3c17282

[5] Kreisel, J., Alexe, M. & Thomas, P. A. A photoferroelectric material is more than the sum of its parts. *Nat. Mater.* **11**, 260–260 (2012). 22437772 10.1038/nmat3282

[6] Yang, S. Y. et al. Above-bandgap voltages from ferroelectric photovoltaic devices. *Nat. Nanotechnol.* **5**, 143–147 (2010). 20062051 10.1038/nnano.2009.451

[7] Huang, H. T. Ferroelectric photovoltaics. *Nat. Photonics* **4**, 134–135 (2010).

[8] Chen, C. S. et al. Emerging 2D ferroelectric devices for in-sensor and in-memory computing. *Adv. Mater.* **37**, 2400332 (2025). 38739927 10.1002/adma.202400332 PMC11733831

[9] Wu, G. J. et al. Author correction: ferroelectric-defined reconfigurable homojunctions for in-memory sensing and computing. *Nat. Mater.* **23**, 723–723 (2024). 38168808 10.1038/s41563-023-01795-8

[10] Cohen, R. E. Origin of ferroelectricity in perovskite oxides. *Nature* **358**, 136–138 (1992).

[11] Ji, D. X. et al. Freestanding crystalline oxide perovskites down to the monolayer limit. *Nature* **570**, 87–90 (2019). 31168106 10.1038/s41586-019-1255-7

[12] Dong, H. et al. Metal Halide Perovskite for next-generation optoelectronics: progresses and prospects. *eLight* **3**, 3 (2023).

[13] Quan, L. N. et al. Perovskites for light emission. *Adv. Mater.* **30**, 1801996 (2018). 10.1002/adma.201801996 30160805

[14] Chen, P. et al. The promise and challenges of inverted perovskite solar cells. *Chem. Rev.* **124**, 10623–10700 (2024). 39207782 10.1021/acs.chemrev.4c00073

[15] Jiang, K. et al. Quantum engineering of non-equilibrium efficient p-doping in ultra-wide band-gap nitrides. *Light Sci. Appl.* **10**, 69 (2021). 33790221 10.1038/s41377-021-00503-y PMC8012702

[16] Jiang, N. L. et al. Plasmonic-enhanced efficiency of AlGaN-based deep ultraviolet LED by graphene/Al nanoparticles/graphene hybrid structure. *Opt. Lett.* **48**, 3175–3178 (2023). 37319055 10.1364/OL.492248

[17] Liu, K. X. et al. Highly reflective Ni/Pt/Al p-electrode for improving the efficiency of an AlGaN-based deep ultraviolet light-emitting diode. *Opt. Lett.* **49**, 4030–4033 (2024). 39008769 10.1364/OL.532520

[18] Mo, Q. H. et al. Highly efficient and ultra-broadband yellow emission of lead-free antimony halide toward white light-emitting diodes and visible light communication. *Laser Photonics Rev.* **16**, 2100600 (2022).

[19] Jin, M. Y. et al. Signal transmission of 4 GHz beyond the system bandwidth in UV-C LED communication based on temporal ghost imaging. *Chin. Opt. Lett.* **19**, 110602 (2021).

[20] Zhang, L. et al. High-performance quasi-2D perovskite light-emitting diodes: from materials to devices. *Light Sci. Appl.* **10**, 61 (2021). 33741895 10.1038/s41377-021-00501-0 PMC7979804

[21] Ricciardulli, A. G. et al. Emerging perovskite monolayers. *Nat. Mater.* **20**, 1325–1336 (2021). 34112976 10.1038/s41563-021-01029-9

[22] Pan, D. X. et al. Deterministic fabrication of arbitrary vertical heterostructures of two-dimensional Ruddlesden–Popper halide perovskites. *Nat. Nanotechnol.* **16**, 159–165 (2021). 33257896 10.1038/s41565-020-00802-2

[23] Zhang, W. C., Hong, M. C. & Luo, J. H. Halide double perovskite ferroelectrics. *Angew. Chem. Int. Ed.* **59**, 9305–9308 (2020). 10.1002/anie.201916254 32168414

[24] Zheng, W. L. et al. Emerging halide perovskite ferroelectrics. *Adv. Mater.* **35**, 2205410 (2023). 10.1002/adma.202205410 36517207

[25] Zhang, Y. et al. Ferroelectricity in a semiconducting all-inorganic halide perovskite. *Sci. Adv.* **8**, eabj5881 (2022). 35138890 10.1126/sciadv.abj5881 PMC10921957

[26] Chen, B. et al. Surface crystallization modulation toward highly-oriented and phase-pure 2D perovskite solar cells. *Adv. Mater.* **36**, 2312054 (2024). 10.1002/adma.202312054 38327173

[27] Liang, C. et al. Two-dimensional Ruddlesden–Popper layered perovskite solar cells based on phase-pure thin films. *Nat. Energy* **6**, 38–45 (2021).

[28] Qi, Z. F. et al. Tailoring phase distribution of quasi-2D perovskites via taurine-assistance enables efficient blue light-emitting diodes. *Small* **20**, 2304821 (2024). 10.1002/smll.202304821 37658498

[29] Kong, L. M. et al. Smoothing the energy transfer pathway in quasi-2D perovskite films using methanesulfonate leads to highly efficient light-emitting devices. *Nat. Commun.* **12**, 1246 (2021). 33623029 10.1038/s41467-021-21522-8 PMC7902836

[30] He, T. W. et al. Reduced-dimensional perovskite photovoltaics with homogeneous energy landscape. *Nat. Commun.* **11**, 1672 (2020). 32246083 10.1038/s41467-020-15451-1 PMC7125147

[31] Noel, N. K. et al. Unveiling the influence of pH on the crystallization of hybrid perovskites, delivering low voltage loss photovoltaics. *Joule* **1**, 328–343 (2017).

[32] Wang, J. Q. et al. Ultrasensitive polarized-light photodetectors based on 2D hybrid perovskite ferroelectric crystals with a low detection limit. *Sci. Bull.* **66**, 158–163 (2021). 10.1016/j.scib.2020.06.018 36654223

[33] Liu, W. et al. Efficient perovskite solar cells fabricated by manganese cations incorporated in hybrid perovskites. *J. Mater. Chem. C.* **7**, 11943–11952 (2019).

[34] Jiang, Y. Z., Wei, J. L. & Yuan, M. J. Energy-funneling process in quasi-2D perovskite light-emitting diodes. *J. Phys. Chem. Lett.* **12**, 2593–2606 (2021). 33689359 10.1021/acs.jpclett.1c00072

[35] Dong, Q. F. et al. Electron-hole diffusion lengths > 175 μm in solution-grown CH_3_NH_3_PbI_3_ single crystals. *Science* **347**, 967–970 (2015). 25636799 10.1126/science.aaa5760

[36] Liu, Z. H. et al. Chemical reduction of intrinsic defects in thicker heterojunction planar perovskite solar cells. *Adv. Mater.* **29**, 1606774 (2017). 10.1002/adma.201606774 28417481

[37] Zhang, Q. et al. High-quality whispering-gallery-mode lasing from cesium lead halide perovskite nanoplatelets. *Adv. Funct. Mater.* **26**, 6238–6245 (2016).

[38] Li, Z. C. et al. Highly luminescent and ultrastable CsPbBr_3_ perovskite quantum dots incorporated into a silica/alumina monolith. *Angew. Chem. Int. Ed.* **56**, 8134–8138 (2017). 10.1002/anie.201703264 28544211

[39] Guo, L. J. et al. A self-powered UV photodetector with ultrahigh responsivity based on 2D perovskite ferroelectric films with mixed spacer cations. *Adv. Mater.* **35**, 2301705 (2023). 10.1002/adma.202301705 37683840

[40] Zhang, Y. J., Wang, J. & Ghosez, P. Unraveling the suppression of oxygen octahedra rotations in *A*_3_*B*_2_*O*_7_ ruddlesden-popper compounds: engineering multiferroicity and beyond. *Phys. Rev. Lett.* **125**, 157601 (2020). 33095620 10.1103/PhysRevLett.125.157601

[41] Ghasemi Hajiabadi, M., Zamanian, M. & Souri, D. Williamson-Hall analysis in evaluation of lattice strain and the density of lattice dislocation for nanometer scaled ZnSe and ZnSe: Cu particles. *Ceram. Int.* **45**, 14084–14089 (2019).

[42] Shi, Y. et al. Bandgap narrowing and piezochromism of doped two-dimensional hybrid perovskite nanocrystals under pressure. *J. Mater. Chem. C.* **11**, 1726–1732 (2023).

