# Supplementary material for: Phase-pure ferroelectric quantum wells with tunable photoluminescence for multi-state optoelectronic applications
Source: Light Sci Appl. 2025 Jun 30;14:228. doi: 10.1038/s41377-025-01874-2 (PMC12209449; doi:10.1038/s41377-025-01874-2)
Supplement: Supplementary file 1 — supplementary information [file 41377_2025_1874_MOESM1_ESM.docx]

**Supplementary Information for**

Phase-Pure Ferroelectric Quantum Wells with Tunable Photoluminescence for Multi-State Optoelectronic Applications

*Rui Sun^1,2#^*, *Yuping Jia^1,2#^, Bo Lai^1,2^, Zhiming Shi^1,2^, Mingrui Liu^1,2^, Weili Yu^1,2^, Ke Jiang^1,2^, Shanli Zhang^1,2^, Shunpeng Lv^1,2^, Yang Chen^1,2^, Xiaojuan Sun^1,2*^ and Dabing Li^1,2*^*

^1^ Key Laboratory of Luminescence Science and Technology, Chinese Academy of Sciences & State Key Laboratory of Luminescence and Applications, Changchun Institute of Optics, Fine Mechanics and Physics, Chinese Academy of Sciences, Dongnanhu Road No. 3888, Changchun 130033, China

^2^ University of Chinese Academy of Sciences, Yuquan Road No. 19, 100049 Beijing, China

Email: sunxj@ciomp.ac.cn; [lidb@ciomp.ac.cn](mailto:lidb@ciomp.ac.cn)

^#^ These authors contributed equally to this study.

**Supplementary Figures and Tables**

**Fig. S1** SEM images of 2, 4 and 8% MnBr_2_ added films.

**Fig. S2** TEM images of MHP films added with different concentration MnBr_2_. (a) The low magnification images. (b) Local magnified images.

**Fig. S3** The XPS of Mn 2p in 6% MnBr_2_ added film.

**Fig. S4** AFM images of pristine (left) and 6% MnBr_2_ added (right) films (1×1 μm^2^).

**Fig. S5** The relative contributions of different n phases in pristine and x=6 films according to the integral intensity of PB peaks in the corresponding TA spectra, respectively.

**Fig. S6** The photos of pristine BA_2_CsPb_2_Br_7_ film (**a**) and 6% MnBr_2_ added BA_2_CsPb_2_Br_7_ film (**b**) with solvent evaporation.


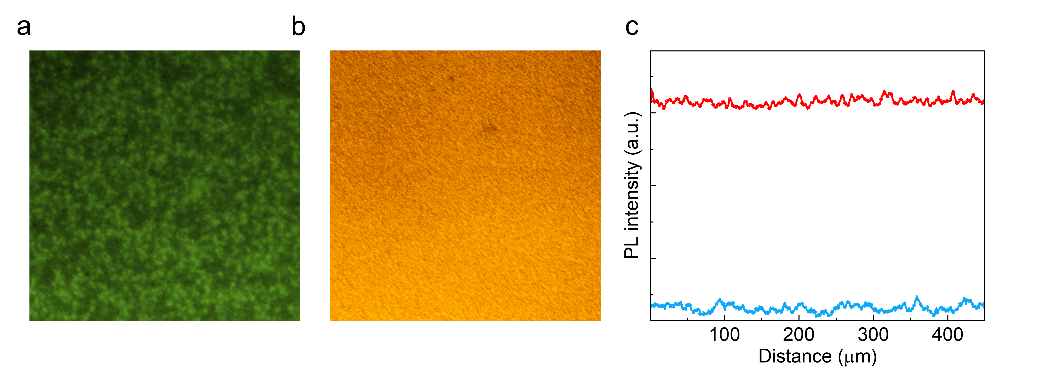


**Fig. S7** The confocal fluorescence microscopy images for pristine (**a**) and 6% MnBr_2_ added (**b**) films and their corresponding PL intensity (**c**).

**Fig. S8** The PLQE measurement results for the 0-8% MnBr_2_ added films, respectively.

**Fig. S9** The PLQE of the 0-8% MnBr_2_ added films.

**Fig. S10** PLQE measurement results for different batches of samples.

**Fig. S11** SCLC measurement results of the electron-only devices (**a, b**) and hole-only devices (**d, e**) based on the pristine and 6% MnBr_2_ added films. The diagram of electron-only (**c**) and hole-only (**f**) device structure.


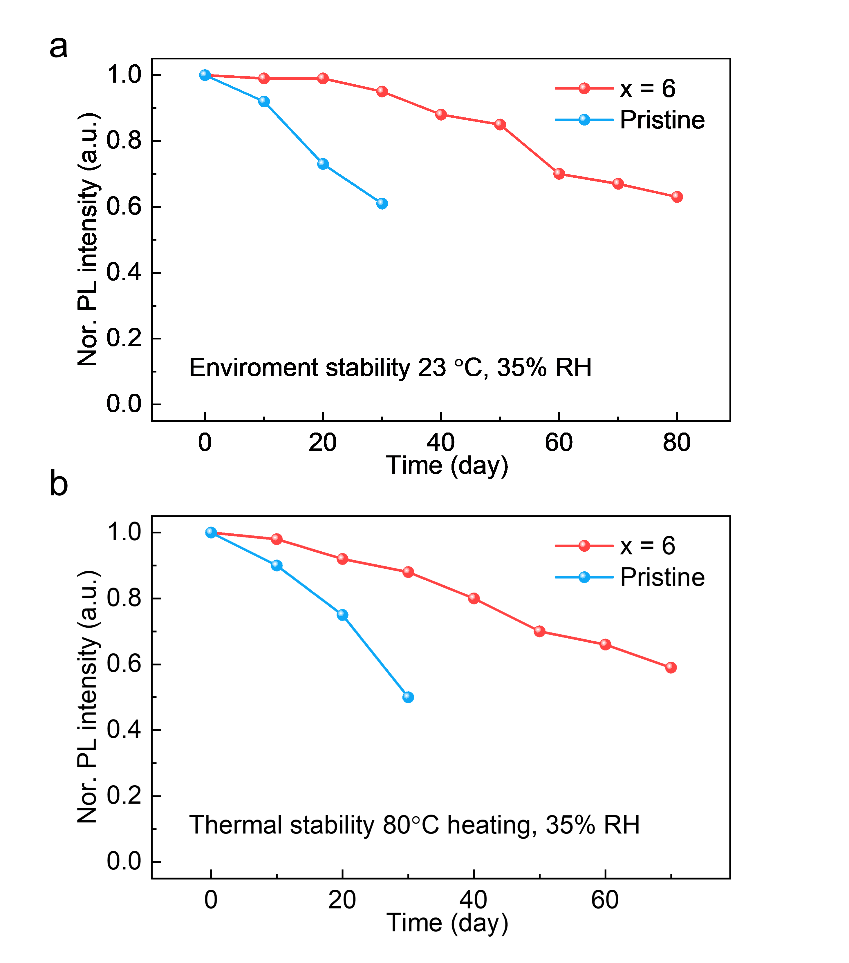


**Fig. S12** Normalized PL intensity of pristine and 6% MnBr_2_ added films exposed to the environment (25 ℃, 30% RH) (**a**) and heated at 80 ℃ in N_2_-filled glove box (**b**), respectively.

**Fig. S13** TRPL spectra of Mn^2+^ in the 2-8% MnBr_2_ added films.

**Fig. S14** P-V hysteresis loop of 6% MnBr_2_ added film.


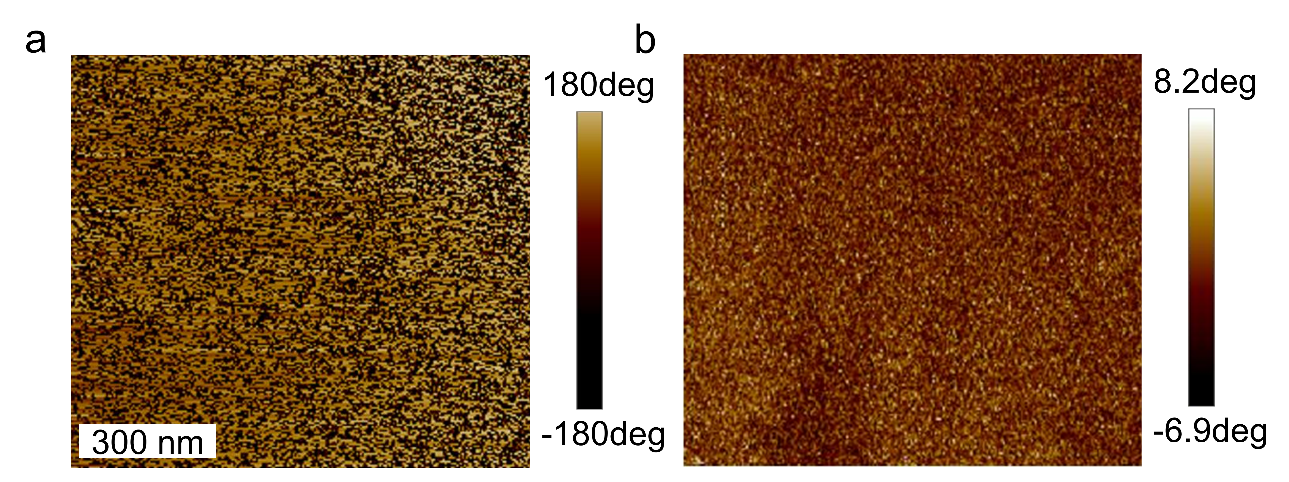


**Fig. S15** Vertical (**a**) and lateral (**b**) PFM images of 6% MnBr_2_ added film, respectively.

**Fig. S16** Vertical PFM phase image after applying a tip voltage V = ±10 V to the film surface.

**Fig. S17** The diagram of voltage dependent PL spectra/PL intensity measurement.

**Fig. S18** PL spectra of 6% MnBr_2_ added film in light modulation device under the loop voltage (0 V → 6 V → 0 V→ -6 V → 0 V).

**Fig. S19** The enhancement factors for orange emission bands as a function of the applied voltage.

The enhancement factor (*f*) is defined as: *f* = s_1_/s_2_, where s_1_ is the integral area of PL spectrum after applying voltage, s_2_ is the integral area of initial PL spectrum without applying voltage.

**Fig. S20** PL decay dynamic curves for ^4^T_1_-^6^A_1_ transition of Mn^2+^ in 6% MnBr_2_ added film under the loop voltage (0 V → 6 V → 0 V→ -6 V → 0 V).

**Fig. S21** XRD pattern of 6% MnBr_2_ added film under the loop voltage (0 V → 6 V → 0 V→ -6 V → 0 V).

**Fig. S22** Five electric/optical dual-modulated nonvolatile PL intensity states: LED off, LED on@0 V, LED on@2 V, LED on@4 V, LED on@6 V.

**Table S1** pH value for DMSO solution added 0-8% MnBr_2_.

| MnBr_2_ concentration | 0% | 2% | 4% | 6% | 8% |
| --- | --- | --- | --- | --- | --- |
| pH value | 7.54 | 7.06 | 6.89 | 5.97 | 5.41 |

**Table S2** Fitting decay components for pristine and 6% MnBr_2_ added films.

| film |  | τ_1_ (a_1_) | τ_2_ (a_1_) | τ_3_ (a_1_) | τ_et_ (a_1_) | $\bar{\boldsymbol{\tau}}$ |
| --- | --- | --- | --- | --- | --- | --- |
| pristine | n = 2 | -0.48 ps  (-22.13) | 4.73 ps  (-22.57) | 45.20 ps  (-6.36) |  | 33.35 ps |
|  | n = 3 | 0.42ps (98.06) | 8.11 ps  (-33.68) | 109.84 ps  (-10.22) | 1.94 ps  (-9.64) | 86.27 ps |
| x = 6 | n = 2 | -0.95 ps  (-112.50) | 4.00 ps  (-37.84) | 33.65 ps  (-8.95) |  | 19.39 ps |
|  | n = 3 | 0.23 ps  (15.97) | 83.86 ps  (-2.12) | 3.50 ps  (-27.98) | 3.50 ps  (13.72) | 47.13 ps |

The curve was fitted by multi-exponential decay convoluted by the time resolution function as^2^:

*f*(t) = *IRF*$\otimes$(($\Sigma_{i}A_{i}e^{-\frac{t-t_{0}}{t_{i}}}+B_{0}ⅇ^{-\frac{t-t_{0}}{t_{i}}}$)H(t-t_0_)) + c

where IRF is the instrument response function:

*IRF*=A_0_$ⅇ^{\frac{-\left( t-t_{0} \right)^{2}}{2\sigma^{2}}}$

𝐴_i_ is the relative amplitude of each exponential decay, 𝐵_0_ is the relative amplitude of the background from excited state absorption.

The triple exponential decay can fit well to all the data in **Figure 3c, d**.

**Table S3** The electron and hole defect densities of the films without and with 6% MnBr_2_ added.

| Sample | V_TFL_ (e) V | V_TFL_ (h) V | Nt (e) cm^-3^ | Nt (h) cm^-3^ |
| --- | --- | --- | --- | --- |
| Pristine | 0.691 | 0.732 | 2.68×10^15^ | 2.84×10^15^ |
| 6% MnBr_2_ | 0.345 | 0.408 | 1.33×10^15^ | 1.53×10^15^ |

**Table S4** Lattice distortion degree fitting results for 6% MnBr_2_ added film under the loop voltage (0 V → 6 V → 0 V→ -6 V → 0 V).

| Applied voltage (V) | 0 | 2 | 4 | 6 | 4 | 2 | 0 | -2 | -4 | -6 | -4 | -2 | 0 |
| --- | --- | --- | --- | --- | --- | --- | --- | --- | --- | --- | --- | --- | --- |
| ɛ E-4 | 0.84 | 1.06 | 5.40 | 7.43 | 7.41 | 6.91 | 7.04 | 2.58 | 1.37 | 5.20 | 4.95 | 5.04 | 4.64 |

In XRD patterns, the broadening of peaks is mainly caused by lattice distortion. W-H method is used to study the variation of lattice distortion with applied voltage^3^:

$\beta\cos\theta=4\varepsilon\sin\theta+\frac{K\lambda}{D}$,

where $\beta$ is the angular line width at half of the maximum intensity, $\varepsilon$ is the lattice distortion of the nanocrystal, λ is the wavelength of X-ray (CuK_α_) radiation, θ is the Bragg diffraction angle, K is the sharp factor, and D is the average crystallite size.

**References**

1 Shen, X. *et al.* Zn-Alloyed CsPbI_3_ Nanocrystals for Highly Efficient Perovskite Light-Emitting Devices. *Nano Letters* **19**, 1552-1559 (2019).

2 Di, H., Xing, Z., Xie, X., Zhao, Y. & Li, B.-H. Effects of the Phase Spatial Distribution on the Intrinsic Carrier Dynamics in Quasi-2D Perovskite Films. *ACS Materials Letters* **6**, 2223-2230 (2024).

3 Ghasemi Hajiabadi, M., Zamanian, M. & Souri, D. Williamson-Hall analysis in evaluation of lattice strain and the density of lattice dislocation for nanometer scaled ZnSe and ZnSe: Cu particles. *Ceramics International* **45**, 14084-14089 (2019).
